# Measuring Patient Similarity on Multiple Diseases by Joint Learning via a Convolutional Neural Network

**DOI:** 10.3390/s22010131

**Published:** 2021-12-25

**Authors:** Sang Ho Oh, Seunghwa Back, Jongyoul Park

**Affiliations:** 1 Research Center of Electrical and Information Technology, Seoul National University of Science and Technology, Seoul 01811, Korea; shoh0320@seoultech.ac.kr; 2 Department of Industrial Engineering, Yonsei University, Seoul 03722, Korea; bsh3069@yonsei.ac.kr; 3 Department of Applied Artificial Intelligence, Seoul National University of Science and Technology, Seoul 01811, Korea

**Keywords:** patient similarity measurement, multiple diseases, feature learning, joint learning, convolution neural network, electronic health records

## Abstract

Patient similarity research is one of the most fundamental tasks in healthcare, helping to make decisions without incurring additional time and costs in clinical practices. Patient similarity can also apply to various medical fields, such as cohort analysis and personalized treatment recommendations. Because of this importance, patient similarity measurement studies are actively being conducted. However, medical data have complex, irregular, and sequential characteristics, making it challenging to measure similarity. Therefore, measuring accurate similarity is a significant problem. Existing similarity measurement studies use supervised learning to calculate the similarity between patients, with similarity measurement studies conducted only on one specific disease. However, it is not realistic to consider only one kind of disease, because other conditions usually accompany it; a study to measure similarity with multiple diseases is needed. This research proposes a convolution neural network-based model that jointly combines feature learning and similarity learning to define similarity in patients with multiple diseases. We used the cohort data from the National Health Insurance Sharing Service of Korea for the experiment. Experimental results verify that the proposed model has outstanding performance when compared to other existing models for measuring multiple-disease patient similarity.

## 1. Introduction

With the ever-growing size and availability of electronic health records (EHRs), many applications on health-related studies become possible to analyze. Patient similarity analysis is of significant interest to researchers because it can be applied to various health research fields, such as precision medicine [1]. It establishes a group of similar patients by measuring the distance between patients and predicts the target patient’s condition by the common phenotype of the similar cohort. With the right patient similarity network built from large-scale data in place, physicians can retrieve a cohort of similar patients for a target patient based on the case of study, make medical comparisons, and, thereafter, make effective personalized treatment plans [2]. Patient similarity analysis is one way to utilize EHRs and facilitate integration from the whole database for data-driven medical decision-making.

EHRs are a complex collection of various medical concepts that can be broadly divided into structured and unstructured data. Unstructured data have no fixed structure, and are usually composed of text, images, and signals. At the same time, structural data are stored in a table, which is more applicable for computer processing than unstructured data. EHRs are also hierarchical, heterogeneous, and sequential. ‘Hierarchical’ means that there may be multiple medical concepts under each observation. For example, multiple medications and multiple procedures may occur under a visit of a patient’s profile. ‘Heterogeneous’ means that the difference between each patient’s profile can be huge, as the frequency of each patient’s visit to the hospital and the medical events under each visit may vary widely. Lastly, sequential means that the patient’s condition changes with time. That is, the observation results are different at different times. These challenges described above often lead to difficulties in utilizing EHRs.

Converting complex, irregular, and sequential EHRs into the appropriate form is the first step in the health-related research by patient data representation. Due to insufficient domain knowledge, statistics are often used to evaluate patient information. For example, the frequency-inverse document frequency (TF-IDF) method can evaluate the importance of clinical concepts in medical records. The importance of the concept increases proportionally with the number of times it occurs in the patient’s medical records, but at the same time decreases, as opposed to the frequency it occurs in other patients’ medical records. Reference [3] used a vector consisting of the international classification of diseases (ICD)-10 codes to represent individual patients, and assigned weights to ICD-10 codes using the TF-IDF method. References [4,5] features were extracted from diagnostic codes, drug components, and lab tests with TF-IDF methods, and similarity was measured using Euclidean distances. However, the above studies did not take into account the sequential properties of obtaining the patient presentation. With the development of deep learning, which is good at longitudinal data processing, several studies have conducted patient presentations using neural networks. Reference [6] used auto-encoder as a deep learning framework for the patient representation. References [7,8] established a vector representation for medical concepts from longitudinal EHRs, and a vector representation of each visit is obtained from the sum of the vectors of medical concepts arising from that visit. This vector representation also considers two levels of embeddings: the medical concept level and the visit level. Not only does this work enhance the effectiveness of the prediction task, but it is also meant to apply the learned interpretable representation to other problems. Reference [9] proposes a model that uses a new time-aware long short-term memory (LSTM) module to process irregularly spaced longitudinal EHRs and obtain effective patient representation by capturing independencies in the sequence.

The purpose of patient similarity is to derive appropriate similarity between patient pairs based on EHRs. Well-measured similarity can be applied to various medical fields, including personalized medicine, disease prediction, and cohort analysis [10,11,12,13,14]. Recently, patient similarity studies based on supervised learning have been conducted to use feedback information from medical professionals. The purpose of supervised patient similarity learning is to learn patient similarity more accurately using expertise as feedback. Reference [15] applies the large-scale machine learning (LSML) algorithm to measure patient similarity and proposes a local supervised similarity learning algorithm. The algorithm identifies homogeneous and heterogeneous neighbors simultaneously, using expert-given labels for each pair of patients as supervised information. Reference [16] proposed a patient similarity framework that introduces group lasso into the objective function to obtain a low-dimensional sparse similarity matrix. This low-ranking mapping avoids the drawbacks of similarity learning algorithms, which have relatively high computational costs. Reference [17] proposed the uncorrelated patient similarity learning framework using maximum likelihood estimation to obtain parameters of similarity functions. In addition, two regular entries are introduced to implement feature selection independent of sparse feature selection. Reference [18] proposed multi-task sparse metric learning to measure patient similarity, a multi-task triple constraint sparse metric learning framework. Optimization is based on triple constraints, which bring similar patients closer and distance other speakers to achieve metric learning.

Recently, studies have been conducted to learn patient similarity measurements and obtain patient presentation considering the temporal properties of longitudinal EHRs. Reference [19] represented the longitudinal EHRs of each patient by a matrix constructed by stacking equal-length embedded vector representations of each visit as the input to the network, which preserves the expression of the temporal characteristics of the longitudinal data. Based on the structure composed of bi-directional convolutional neural networks (CNN) and supervised metric learning, a matching matrix is used to calculate the similarity between the two embedded vectors derived from the deep network. Reference [20] proposed a time-fusion CNN framework that preserves the local temporal relationship and obtains a global contribution of different time intervals for patient similarity measurement. The work also made predictions for target patients according to the phenotype of the k-nearest patients. Reference [21] proposed a CNN-based triple patient similarity learning framework to learn a margin that separates similar and dissimilar patients better.

However, most similarity measurement studies have only studied single or specific diseases. It is not realistic to consider only one specific disease, and it ignores the characteristics of the accompanying disease. Therefore, a similarity measurement study for patients with multiple diseases is needed. Recently, there are studies dealing with multiple diseases patient similarity [22,23], but still need more participation from the researchers. To measure the similarity of patients with multiple diseases, we proposed a CNN-based model that jointly learns identity representation and measures the similarity of patients with multiple diseases. The proposed model consists of two parts. The former is feature learning, which classifies the input data as having any disease and gives them an identity presentation. The latter part is similarity learning, which measures similarity between patients. We trained these two parts simultaneously to measure the similarity of patients with multiple diseases. Furthermore, we compared the similarity score between our proposed ‘joint learning’ model with feature learning and similarity-based learning models to validate the performance. Moreover, we verified the performance by classification evaluation metrics to test the performance of proposed joint learning.

## 2. Materials and Methods

This section explains how the patient’s demographic information, diseases, and prescription was represented and how to measure the similarity between patients with multiple diseases. Lastly, a CNN-based model, which applies feature learning and similarity learning, was introduced. All clinical processes were discussed and validated by a doctor from Seoul Red Cross hospital in Seoul, South Korea.

### 2.1. Patient Record Representation

**Figure 1:**
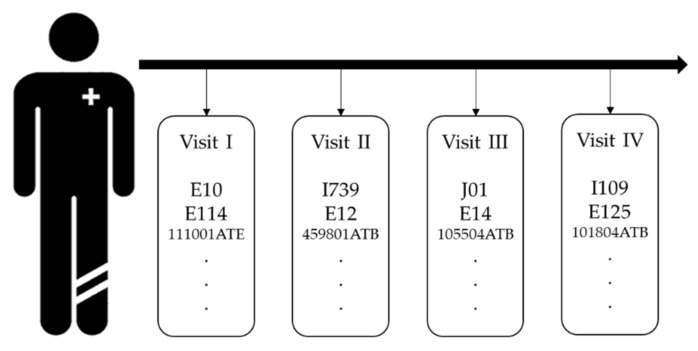
An Illustration of EHRs.

The purpose of this section is to define the input to the model. As shown in Figure 1, raw EHRs are sequential and irregular. We need to transform the raw EHRs to numeric form to process through our proposed model. The most basic method is to use one-hot encoding. Since EHRs are longitudinal data, the temporal property should be considered when obtaining patient representation; therefore, we used matrix to represent the patient’s medical record, which was obtained by stacking the same length of embedded vector for each visit. In our research, each visit contains demographic information, such as gender and age group, diagnosis codes, and prescription codes.

Through Doc2Vec, we can obtain the visit level embedding vector and same dimensional vector representations for patients’ historical records [24]. The patient p can be represented by a matrix P with size e×v, which e is the embedding size and v is the number of visits. We used paragraph vector with the distributed memory (PV-DM) model of Doc2vec to obtain patient representations, used demographics, disease, and treatment information from EHRs, and compared the embedding dimensions by setting 128 and 256. We confirmed that the patient’s representation performance was better at 128 dimensions and that each patient’s visit was converted to 128 dimensions vector. Furthermore, to equalize the number of visits to every patient, we set the maximum number of visits to 200, and applied zero padding. Consequently, a patient representation matrix of 200 × 128 were obtained.

### 2.2. Self-Attention Module

The objective of the attention mechanism is to select more important information from that task, and it is derived from the human visual attention mechanism. Restricted attention to capturing useful information significantly improves efficiency and accuracy. Attention mechanisms combined with CNN and recurrent neural networks (RNN) structures have achieved superior performance in natural language processing and computer vision.

In our research, 200 × 128 size matrix representation of the patient history records can be converted into a vector by convolution and the pooling layer through the above method. However, although the basic convolution operation can capture the temporal information, there is a limitation that the information is equally treated during this process. To further improve the ability of patient vector representation, we introduce an attention mechanism in the convolutional layer in the network.

### 2.3. Patient Labeling for Similarity Learning

#### 2.3.1. Feature Learning Label

We considered three diseases—diabetes, cerebrovascular disease, and ischemic heart disease, which may cause cascaded disease occurrence to each other. We used feature learning to obtain the identity representation of patients embedded with a matrix, and feature learning needs a label for supervised learning. We considered patients’ age, gender, blood pressure (BP), body mass index (BMI), and disease codes, and then those factors are represented as one-hot encoding. For continuous measurements, such as age, blood pressure, and BMI, we divide them into three groups, to express with one-hot encoding, as shown in Table 1.

#### 2.3.2. Similarity Learning Label

Similarity learning measures similarity based on distance and needs a label as supervised learning. We use a patient’s age, gender, BP, BMI, and disease information as the patient’s label to measure the similarity. We generate similarity labels by comparing feature categories among seven categories, (age, gender, BP, BMI, diabetes, cerebrovascular disease, ischemic heart disease) if there are more than equal to four categories (median of 7) match between patients, we label them “1”, as similar patients, otherwise “0”, as shown in Table 2.

### 2.4. Patient Similarity Measurement

#### 2.4.1. CNN Based Framework

CNN has superior advantages in feature extraction due to its strong representation capabilities. Feature extraction is performed on local information via convolutional operations. Complete information can be obtained by integrating local information. The feature detection layer of CNN avoids explicit feature extraction, but implicitly learns from the training data. Furthermore, the complexity of the network is reduced due to the sharing of local weights, which is also a significant advantage of convolutional networks for fully connected networks.

This section will show how to obtain the embedded vector representation of a patient’s history matrix via a CNN structure. Feature extraction is first performed via convolutional layers. Our goal is to explore relationships between accesses in the patient matrix, so we use 1D filters to perform convolutional tasks on the access dimensions of patient matrix representations. More specifically, information from matrix history maps is extracted using different filters, and the parameters of each filter are obtained through network optimization.

For each filter, $w\in {\mathbb{R}}^{h\times e}$*,* where h represents the size of the filter, which is the number of consecutive visits used to generate features in the convolution operation. Where e is the embedded dimension for each access defined in the previous work. By performing a feature extraction with temporal meaning through a convolution operation, we have Equation (1), where * represents the convolution operation, b∈ℝ represents the error term, $P_{i:i+h-1}$ represents the concatenation of the $i^{th}$ to the ${\left(i+h-1\right)}^{th}$ visit vectors, and the result $c_{i}$ represents a feature obtained after the convolution operation of $P_{i:i+h-1}$.

(1) $$c_{i}=ReLU\left(w*P_{i:i+h-1}+b\right)$$

Applying the filter to the entire patient matrix representation with a stride of 1, we can obtain v+h−1 features, where v is the number of visits. Through these features, we can get a feature map c as Equation (2).

(2) $$c=\left[c_{1},c_{2},c_{3},\ldots ,c_{v-h+1}\right]$$

The pooling layer reduces the obtained feature map. Here, we use max pooling, and the purpose is to extract the most essential information in the feature map to get a reduced feature map, which can reduce the computational complexity and make the feature representation more robust and avoid overfitting. We obtained a feature map through convolution operations of the data of embedded patients represented by matrix. We used Conv1D because our data have sequential properties. In addition, Maxpooling1D was used to obtain the largest value while reducing dimensions on the obtained feature map. Since our EHRs has sequential features, we added a self-attention module to effectively extract information based on its sequential features and patient historical records. A CNN block computes Conv1D, Maxpooling1D, and self-attention, in order. Finally, three CNN blocks result in vector presentation of the patient.

**Figure 2:**
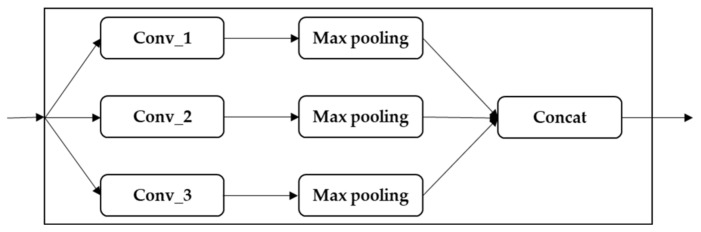
CNN block.

In each CNN block, as shown in Figure 2, three convolutional operations and pooling operations are performed on the input feature map in a parallel manner. All of the output is joined into a relative deep feature map. Because different convolution operations and pooling operations can obtain additional information of the input feature map, parallel, conducting these operations, and combining all the outputs, will get better feature representation. The different convolutional layers aim to extract various features, and max-pooling layers are used to reduce the intermediate representation. Then, the feature maps derived from different branches are concatenated together as a feature map for the following step processing.

#### 2.4.2. Feature Learning

**Figure 3:**
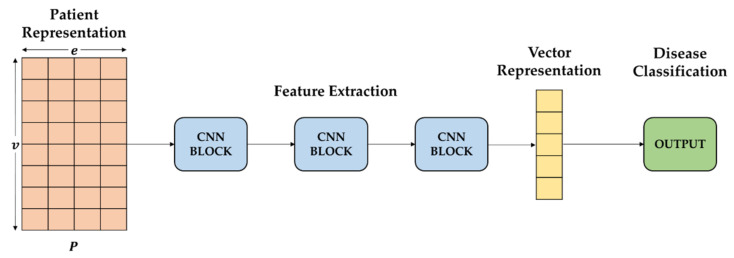
An Illustration of Feature Learning.

We employ a sigmoid classifier on top of the base network for feature learning to learn patient identity representation [25]. With a patient embedded matrix into the CNN network, it extracts patient information. Moreover, identity presentation is obtained through feature learning. Feature learning labels are used to supervise the categorization training. We use sigmoid to obtain probability values for each label’s output to classify the patient’s disease and then use binary cross-entropy as a loss function as Equation (3).

(3) $$L^{classify}\left(y,\tilde{y}\right)=-\underset{i}{\sum }\left[y_{i}*\log\tilde{y_{i}}+\left(1-y_{i}\right)*\log\left(1-\tilde{y_{i}}\right)\right]$$

where y represents the true label and $\tilde{y}$ represents the output of the network. The $y_{i}$ represents the $i^{th}$ label in the multiple labels and the ${\tilde{y}}_{i}$ indicates the $i^{th}$ output probability that the patients pairs share the $i^{th}$ disease. The feature-learning model is shown in Figure 3.

#### 2.4.3. Similarity-Based Learning

Appropriate metrics derive the patient similarity. With a patient embedded matrix into the CNN network, it extracts the patient’s information. Thus, two vectors are obtained. Both vectors are 800-dimensional vectors, which obtain 800-dimensional vectors through vector-based Euclidean distance calculations. To determine whether the patient is similar to another patient, we measure the similarity by the contrastive loss function. This loss function learns to be similar if the values from the distance calculation of both data are small (contrarily, if the values are large). Thus, ultimately, if we calculate contrastive loss, each disease results in a similarity score. We compare similarity measurement performances with this similarity score.

We adopt contrastive loss in [26] as a loss function to measure similarity after computing the vector-based Euclidean distance. This loss function Equation (4) was applied for each disease. Y is a binary label with 1 if the two inputs $\left(X_{i}^{a},X_{i}^{b}\right)$ are similar, otherwise 0. $e_{i}$ is a vector-based Euclidean calculation of two feature representations extracted from input data via CNN. This loss function learns that if the inputs are similar, the following formulations are removed to make the values smaller, and if they are not similar, the preceding formulations are removed to make the values bigger.

(4) $$L^{similarity}\left(e_{i},{\left(Y,X^{a},X^{b}\right)}_{i}\right)=\left(Y\right)\frac{1}{2}{\left(e_{i}\right)}^{2}+\left(1-Y\right)\frac{1}{2}{\left\{\max\left(0,margin-e_{i}\right)\right\}}^{2}$$

### 2.5. Joint Similarity Learning Architecture

**Figure 4:**
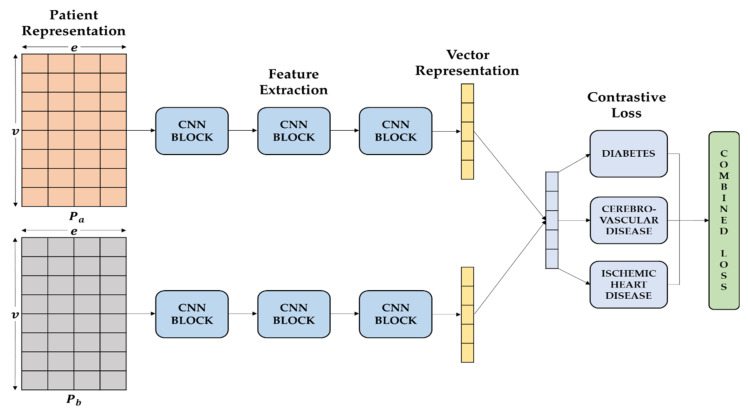
An illustration of proposed similarity learning.

Figure 4 shows joint learning structure transforms embedded matrix representations containing temporal information from patient records into vector representations and learns two methods simultaneously through transformed vector representations, which we draw ideas from [27]. The feature extraction part of this CNN-based network is learned by sharing weights. For each CNN block, we perform convolutional operations to extract the input features and use a max-pooling layer to reduce the intermediate representation. We then introduce a self-attention module for each CNN block to improve the ability of the representation. Finally, we classify what diseases the patient has, and then learn the similarity for each disease after calculating the distance of the two vectors, and optimize them by summing their respective loss functions Equation (5).

(5) $$L^{total}=L^{classify_{a}}+L^{classify_{b}}+L^{similarity}$$

The algorithm of joint learning is shown as Algorithm 1.
**Algorithm 1** Joint Learning**Input**: P: patient data, D: Doc2vec matrix, L: dataset true labels**Output**: patient similarity score on test dataset **for** i in dataset **do**  **let**
$P_{i}$ be the patient matrix of i  **for** j in i **do**   $V_{j}$ <- vectorize(j,D)   **append**
$V_{j}$
$\;\mathrm{to}\;P_{i}$
 $P_{train}$$,\;P_{test}$$,\;L_{train}$$,\;L_{test}$ <- split train subset and test subset  S <- CNN Joint Learning$([P_{train\_a}$$,\;P_{train\_b}$$],[L_{train\_a}$$,\;L_{train\_b}$]) score <- evaluate $(i,\;[L_{test\_a},L_{test\_b}$], S) **return** score

### 2.6. Data Descriptions

**Figure 5:**
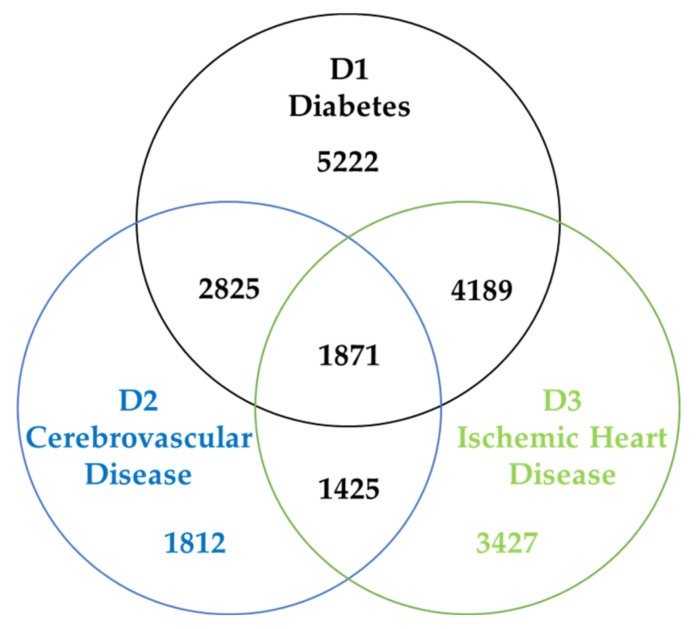
Population distribution of diabetes, cerebrovascular disease, and ischemic heart disease.

The data are provided by National Health Insurance Service of Korea and covers 12 years of EHRs. We filter the visit history related to diabetes (ICD-10 codes of E10-E14), cerebrovascular disease (ICD-10 codes of I61-I69), and ischemic heart disease (ICD-10 codes of I20-I25). The description of the collected dataset is shown in Table 3 and Figure 5.

According to the patient’s diagnostic information, classify them in the order of visits with the same patient identification number, and then extract diagnostic information, demographics, to form an EHR set. Since extreme data can bias, the 99th quartile of each medical event is checked, and the data are removed if they have more than 12 diagnostics, and 200 visits.

### 2.7. Handling Data Imbalance

To deal with the data imbalance issue, we applied the data augmentation for imbalance multi-label data, which is known as multi-label synthetic minority over-sampling (MLSMOTE) [28]. MLSMOTE is one of the most popular and effective data augmentation techniques in the case of multi-label classification. As the name suggests, it is an extension or variant of the synthetic minority over-sampling technique (SMOTE) [29]. In SMOTE, we provide data and augment it to generate more samples of the same class from which the reference point has been chosen, but it failed in a multi-label situation because the instances of the data had many labels attached with it. Therefore, there is a chance that a sample with a minority label could also have a majority label, so we will have to construct labels for the synthetic data as well. Labels in the majority were referred to as head labels, while labels in the minority were referred to as tail labels in multi-label contexts [30].

### 2.8. Model Parameters

First, model training allows us to obtain optimized parameters in our network. Our deep learning parameters are set as follows: the width of the convolution filter is 3, and the number of convolution filters takes on 128, 64, and 32. Max-pooling and self-attention modules are added after each convolution operation. We added a dropout normalization with a dropout rate of 0.3 after the max-pooling layer to overcome the overfitting problem. In the feature extraction part, the last feature extraction layer, where the vector representation size was 800. The batch size was 128 and epochs with 100, and the early stop was used to prevent overfitting. Moreover, 100,000 patient pairs as experimental data with the ratio of training and test sets was 8:2. The performance of the test set proves that our model can improve the ability of patient similarity learning. The model was implemented by TensorFlow-Keras and optimized by Adam [31].

## 3. Results and Discussions

### 3.1. Validation of Proposed Joint Learning Performance

To validate the performance of joint learning, we compared the performance according to each learning approach: similarity-based learning and joint learning. Both models were trained based on CNN. To test the performance of similarity-based learning, we measured similarity, except feature learning. We measured the similarity between every combination of two diseases and three diseases. The results of similarity-based learning are shown in Table 4.

Similarity-based learning measures similarity without feature learning and learns without identity presentation. The maximum similarity scores of each combination of diseases seem to be high, but the mean of similarity scores are below 85%. To validate the impact of joint learning, we showed the similarity score results in Table 5.

The similarity score of joint learning increased by 0.084 on average than similarity-based learning. Moreover, the mean of the similarity score of every combination exceeded 90%, except the diabetes and cerebrovascular disease combination, but almost reached 90%. These results verified that feature learning effectively expresses patient information. Therefore, our proposed joint learning improved measuring patient similarity with multiple diseases.

### 3.2. Validation of Joint Learning Model by Classification Evaluation Metrics

**Figure 6:**
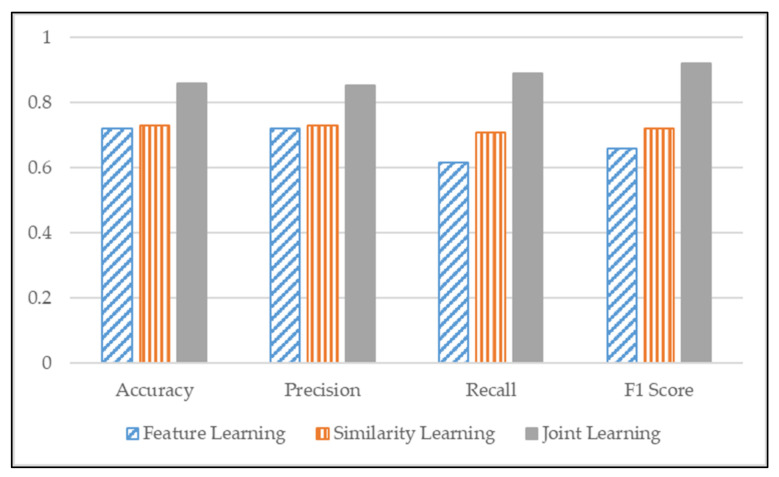
Performance of feature learning, similarity learning joint learning.

This section compares the performance of three different learning approaches: feature learning, similarity-based learning, and joint learning by classification evaluation metrics. In feature learning, we follow the procedure of base CNN network training as a categorization classifier. Similarity-based learning is a model that removes the categorization classifier. Finally, joint learning is a method of combining the two models. Since we transform the similarity learning problem into a supervised binary classification problem, we compare the proposed model with feature learning and similarity learning performance in binary classification tasks. We selected accuracy, precision, recall, and F1 scores as performance metrics from classification evaluation. Classification metrics are calculated from true positives, false positives, false negatives, and true negatives. Accuracy is one metric, which gives the fraction of predictions our model got right. Precision gives the fraction of correctly identified as positive out of all predicted as positives. Recall gives the fraction that correctly identified as positive out of all positives. Lastly, the F1 score is the harmonic mean of the model’s precision and recall because it is not sensitive to extremely large values, unlike simple average [32]. The results are shown in Figure 6.

In Figure 6, as we can observe, our proposed joint learning model performed better in accuracy, precision, recall, and F1 score, about 20%, than applying feature learning and similarity-based learning. With these results, we validated that using feature learning alone does not measure similarity properly. Moreover, by using similarity-based learning alone, it poorly classifies embedded data for each patient.

### 3.3. Comparison between Existing Similarity Measurement Algorithms

To validate the performance of the proposed joint learning algorithm, we include another four existing distance metric algorithms for comparison purposes. The considered existing algorithms are Euclidean distance, Cosine similarity, Mahalanobis distance [33], and localized supervised metric learning (LSML) [34]. The performance comparison results by classification evaluation metrics of five algorithms are shown in Table 6.

The Euclidean distance and Cosine similarity algorithms are basic metrics for measuring patients’ similarity. Those algorithms directly measure similarity without learning parameters and, therefore, the results are low. Moreover, Mahalanobis and LSML results are higher than Euclidean and Cosine, but as we can observe, the proposed joint learning algorithm has the highest scores among all algorithms.

## 4. Conclusions, Limitations, and Future Works

Patient similarity measurement research is a fundamental study for application in various medical fields. Appropriate similarity measurements are required for application to other healthcare areas. Patients’ historical records, particularly those of patients with chronic diseases, rely heavily on temporal information. It is important to obtain a vector representation of the patient’s historical records, with temporal properties to capture information in the historical patient records, and measure the similarity between patients. Patient similarity learning is based on learning a metric matrix that may effectively quantify the similarity between patients using paired constraints. Compared to the primary distance metric, patient similarity learning can change the patient representation into a new characteristic space based on the learned matching matrix, resulting in closer distances between similar patients and further distances between different patient samples.

Existing studies measure the similarity of single diseases well, but measuring similarity with other diseases still needs more research. To address the limitations of existing similarity studies that consider only one disease, we propose a model to measure multiple disease similarities by jointly combining feature learning and basic similarity learning. This research verified that our proposed joint learning model improves the similarity measurement performance of multiple diseases by over-performing among other existing similarity measurement algorithms. Therefore, we validated that the proposed model is suitable for measuring the patient similarity of multiple diseases.

The limitation of our research is the population of the cohort database because we only dealt with one kind of medical database, which the National Health Institute of Korea provides. As shown in Figure 5, there are only a few records of diabetes, cerebrovascular disease, and ischemic heart diseases. For those three diseases, as well as other diseases, there are an unsatisfying number of records available, unless the disease is general, such as hypertension. It was challenging to find insight and to measure similarities between patients with the small dataset.

In future works, we would like to search and acquire other electronic health records by collaborating with the research center of hospitals in Korea or databases from other countries, such as Medical Information Mart for Intensive Care (MIMIC)-III to expand the population number and verify our proposed model. Moreover, we will consider more appropriate and adequate variables, such as more specific body check measurements to strengthen our concept of the similarity measurement. Furthermore, we will extend our research to disease prediction and precision medicine by applying our similarity measurement model to broaden the research on healthcare applications.

## Figures and Tables

**Table 1:** Group division for continuous measurement.

	Age	BP	BMI
Group 1	0–30	Prehypertension	Underweight
Group 2	31–60	Stage 1	Normal
Group 3	Over 61	Stage 2	Overweight

In BP, the prehypertension range is 120–139 mmHg of systolic blood pressure (SBP) and 80–89 mmHg of diastolic blood pressure (DBP). Stage 1 range is SBP of 140–159 mmHg and 90–99 mmHg of DBP. Lastly, the range of stage 2 is SBP over 160 mmHg and DBP over 100 mmHg. In BMI, the underweight group range is below 18.5 kg/m^2^. Normal group range is between 18.5 kg/m2 to 22.9 kg/m^2^. The overweight group is composed of BMI more than 23 kg/m^2^.

**Table 2:** Example of similarity labeling.

Features		Patient A	Patient B	Match	No. of Match
Age	0–30	0	0	0	4
	31–60	1	1	1	
	Over 61	0	0	0	
Gender	Male	1	1	1	
	Female	0	0	0	
BP	Prehypertension	0	0	0	
	Stage 1	1	0	0	
	Stage 2	0	1	0	
BMI	Underweight	0	0	0	
	Normal	0	0	0	
	Overweight	1	1	1	
Disease	Diabetes	1	0	0	
	Cerebrovascular disease	1	1	1	
	Ischemic heart disease	0	0	0	
Output	Similarity Label	1			

If the patient’s record belongs to a certain feature, we write 1, otherwise 0. In the match column, if the features of patients A and B match, we write 1, otherwise 0. Similarity label will be 1 if the number of matches is more than equal to 4, otherwise it is 0.

**Table 3:** Dataset descriptions.

Descriptions	Male	Female
Sex (%)	56	44
Age, mean(SD)	57.8 (23.4)	60.5 (21.5)
SBP (mmHg), mean (SD)	126.4 (27.1)	131.8 (24.8)
DBP (mmHg), mean (SD)	78.8 (12.6)	81.8 (14.2)
BMI (kg/m^2^), mean (SD)	22.3 (5.8)	23.1 (4.2)
No. of visits	1,052,085	648,066

**Table 4:** Similarity-based learning results.

	Diabetes and Cerebrovascular Disease	Diabetes and Ischemic Heart Disease	Cerebrovascular Disease and Ischemic Heart Disease	Diabetes and Cerebrovascular Disease and Ischemic Heart Disease	Average
Mean	0.7859	0.8027	0.8429	0.8474	0.8197
Max	0.8757	0.8607	0.8952	0.9274	0.8897
Min	0.5571	0.7470	0.8035	0.8137	0.7304

The metric presented in Table 4 is the similarity score calculated by Equation (4). The range of the score is between 0 and 1. If the score is closer to 1, it means higher similarity; otherwise, it is closer to 0. The mean, max, and min came from the score of patient pairs.

**Table 5:** Joint learning results.

	Diabetes and Cerebrovascular Disease	Diabetes and Ischemic Heart Disease	Cerebrovascular Disease and Ischemic Heart Disease	Diabetes and Cerebrovascular Disease and Ischemic Heart Disease	Average
Mean	0.8446	0.9148	0.9227	0.9333	0.9039
Max	0.9589	0.9789	0.9849	0.9782	0.9752
Min	0.6439	0.8125	0.8526	0.8645	0.7934

The metric presented in Table 5 is the similarity score calculated by Equation (5). The range of the score is between 0 and 1. If the score is closer to 1, it means higher similarity; otherwise, it is closer to 0. The mean, max, and min came from the score of patient pairs.

**Table 6:** Performance comparison between existing algorithms.

Algorithms	Accuracy	Precision	Recall	F1 Score
Euclidean	0.5423	0.5218	0.6285	0.5457
Cosine	0.5751	0.5684	0.6071	0.5895
Mahalanobis	0.6573	0.6817	0.7825	0.7782
LSML	0.8015	0.8148	0.8533	0.8759
Joint Learning	0.8572	0.8511	0.8925	0.9227

## Data Availability

The datasets generated and/or analyzed in the current study are not publicly available due to patient information collected by the National Health Insurance Sharing Service, which requires payment for access. However, sample data are available from the corresponding author upon reasonable request.
